# The Antioxidant Activity of a Commercial and a Fractionated Phycocyanin on Human Skin Cells In Vitro

**DOI:** 10.3390/molecules27165276

**Published:** 2022-08-18

**Authors:** Roberto Puglisi, Elisa Biazzi, Daniela Gesmundo, Roberta Vanni, Aldo Tava, Silvia Cenadelli

**Affiliations:** 1 Istituto Sperimentale Italiano Lazzaro Spallanzani, Loc. La Quercia, 26027 Rivolta d’Adda (CR), Italy; 2 CREA Research Centre for Animal Production and Aquaculture, Viale Piacenza 29, 26900 Lodi, Italy; 3 Advanced Cosmetic Science, Department Chemistry “Giacomo Ciamician”, Alma Mater Studiorum Università di Bologna, Via S. Giacomo 11, 40126 Bologna, Italy

**Keywords:** cell culture, color cosmetics, skin barrier, UVA, photoaging, ROS, *Arthrospira platensis*, Spirulina

## Abstract

The protective effects for cells against chemical and UVA stress of a commercial phycocyanin (PC) for food use and a PC extracted from *Arthrospira platensis* (Spirulina) in phosphate buffer were assessed. The purity of the commercial PC, spectrophotometrically estimated as A_620_/A_280_ and confirmed by HPLC, was higher than that of the fractionated PC (2.0 vs. 1.5) but was twofold less concentrated. The oxygen radical antioxidant capacities (ORACs) of the commercial and fractionated PCs were 12,141 ± 1928 and 32,680 ± 3295 TE/100 g, respectively. The degradation of PCs upon exposure to UVA was spectrophotometrically estimated, and cytotoxicity was evaluated with the MTS [3-(4,5-dimethylthiazol-2-yl)-5-(3-carboxymethoxyphenyl)-2-(4-sulfophenyl)-2*H*-tetrazolium) test on human fibroblasts and keratinocytes. A lower level of reactive oxygen species (ROS) was recorded in the two cell lines incubated with the commercial PC after menadione treatment (*p* < 0.01) and UVA exposure (*p* < 0.001) on fibroblasts after 5 min and keratinocytes up to 25 min, compared with controls. Differently, the fractionated PC was not protective and showed significant (*p* < 0.01) paradoxical prooxidant effects. Overall, the PC for food consumption demonstrated a high safety threshold and antioxidant ability to cells that, along with its coloring power, make it an excellent candidate for cosmetic formulations.

## 1. Introduction

Phycocyanin (PC) is a blue, water-soluble, pigment-protein complex belonging to the family of phycobiliproteins, the most relevant group of pigments in cyanobacteria for the adsorption of light energy [1]. Among the various species, PC is synthesized at a very high yield by the photosynthetic cyanobacteria *Arthrospira platensis*, commonly known as Spirulina. Due to its bright color and very low toxicity, it is highly appreciated as a natural food coloring [2]. In addition these features, the growing interest in this molecule stems from its various biological activities, such as antioxidant, anti-inflammatory and anticarcinogenic effects [3]. Based on these properties, PC is increasingly also known and appreciated as a promising cosmetic additive [4].

With regard to cosmetics, the synergistic effect of Spirulina and UV filters was investigated for the development of stable and effective sunscreens [5]. The results demonstrated that a formulation containing UV filters with raw Spirulina algae and a synthetic γ-tocopherol analogue with high antioxidant property significantly improved skin pigmentation and net elasticity after 84 days of treatment compared with the sunscreen alone, in addition to not being an irritant to the skin, according to sensorial analyses. However, the study did not clarify the individual contributions of the two antioxidants, and additional investigation at the cellular level is required to develop more effective and safer sunscreens formulated with PC.

With regard to solar radiation, given their highly damaging nature, UVBs (290–320 nm) have received greater attention than the less energetic component of sunlight, namely UVA (320–400 nm). However, it is now increasingly evident that UVA radiation plays a predominant, and underhanded, role in the damage caused to the skin [6]. In fact, as well as being more abundant in terms of quantity and time during the day, UVAs penetrate deeper than UVBs into the skin, inducing alterations of the dermal connective tissue. These alterations, mainly caused by the massive induction of reactive oxygen species (ROS), are the cause of the onset of skin cancers and of so-called photoaging [7]. For this reason, there is a growing interest in both the medical and cosmetic sectors in functional innovative molecules with radical scavenging properties.

In addition to PC, Spirulina contains vitamins, minerals, unsaturated fatty acids, carotenoids and other nutrients, and as such, it has long been a natural ingredient in nutraceuticals as a dietary supplement [8]. Therefore, along with the increasing commercial interest in and fields of application of Spirulina, strategies have multiplied for improving quality while reducing the costs of microalgae production [9] and the extraction of their molecules of interest [10]. In fact, several methods for the extraction of molecules from cyanobacteria and purification procedures have been described to obtain PC of different grades of purity [11]. According to general rules, PC is considered food-grade when the ratio between the optical density relative to the absorption peak of PC (A_620_) and that of total proteins (A_280_) is ≤0.7; reagent-grade when the ratio is between 0.7 and 3.9 and analytical-grade when it is ≥4.0 [12]. Based on this general classification that drives the economic aspects, increasing numbers of strategies are being proposed for producing low-cost algal matrices that, together with the use of inexpensive extraction methods, make alternative ways of supplying PC more feasible to commercial channels [13]. Otherwise, as demonstrated in a specific economic analysis, recovering high-value molecules other than PC from Spirulina is not so advantageous [10]. However, for a more exhaustive evaluation of the economic and functional aspects of low-cost PCs recoverable from the market with respect to those of more certain origin produced autonomously, more in-depth studies are required. In particular, the majority of studies on the properties of PC are always based on molecules of high purity and of considerable cost, so that the potential of commercial low-grade PC for use in cosmetics is mostly unconsidered [14]. Furthermore, despite their ecological value and natural origin, the bioactive compounds derived from microalgae may comprise either beneficial or toxic constituents [15]. Therefore, further investigation is advised in order to integrate these molecules into increasingly useful and safe end products [16].

The aim of this work was to apply an in vitro cell-based model to assess the antioxidant properties against chemical and physical (UVA) stress of a commercial PC intended for food consumption and those of a PC extracted using a solvent-free method in view of potential enhancements in cosmetics.

## 2. Results

### 2.1. Fractionation and Characterization of PCs

**Figure 1:**
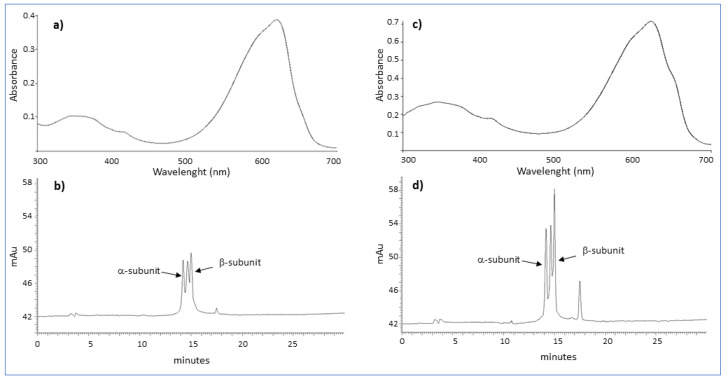
(**a**) Absorption spectrum and (**b**) reverse-phase HPLC profile of a commercial phycocyanin (PC) and (**c**) absorption spectrum (**d**) and reverse-phase HPLC profile of a phycocyanin (PC) fractionated from *Arthrospira platensis*. The two α and β-subunits of PC are reported.

Extraction, precipitation and dialysis ensured an approximate yield of 13% (*w*/*w*) of PC from lyophilized Spirulina biomass. The purity of the two PCs assessed by spectrophotometer (A_620_/A_280_) was estimated as 2.0 for the commercial and 1.5 for the fractionated PCs, corresponding to a reagent-grade classification for both the specimens. Absorption spectrum analysis, showing a maximum absorption peak at 620 nm for both PCs, revealed an absorbance of 0.38 for the commercial (Figure 1a) and 0.70 for the fractionated PCs (Figure 1c), indicating an approximately two-fold concentration of the fractionated PC with respect to the commercial one. 

The reverse-phase HPLC characterization showed four peaks with two specific peaks detected at the time of 14.19 and 15.00 min for both the PCs (Figure 1b,d) that according to literature data [17,18] correspond to the α- and β-subunits of PC. Therefore, this assay also confirmed commercial PC to be approximately two-fold diluted with respect to the fractionated PC.

### 2.2. ORAC Assay

The ORAC evaluation was performed to estimate the antioxidant potential of the commercial and fractionated PCs. Both PCs showed that the 0.1 mg mL^−1^ concentration was contained within the linearity range of the Trolox reference standard curve. That is, ORAC values of the commercial and fractionated PCs were calculated as 12,141 ± 1928 and 32,680 ± 3295 TE/100 g, respectively.

### 2.3. PC Photodegradation

**Figure 2:**
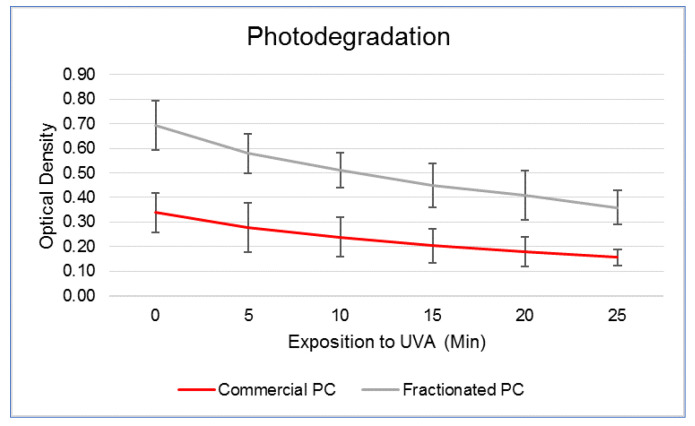
Spectrophotometric readings performed at 620 nm (mean ± SD) at increasing exposure times under a UVA lamp of a commercial phycocyanin (PC) and a PC fractionated from *Arthrospira platensis*.

The spectrophotometric readings carried out on the two PCs at increasing exposure times are shown in Figure 2. Based on the trend, it was calculated that at 25 min exposure to UVA, the commercial and fractionated PCs underwent degradation of 46% and 52%, respectively. Therefore, the evaluation of ROS upon UVA irradiation and related protective efficacy of PCs was carried out within this time frame at 0, 5, 10, 15, 20 and 25 min exposure.

### 2.4. Cytotoxicity Test

**Figure 3:**
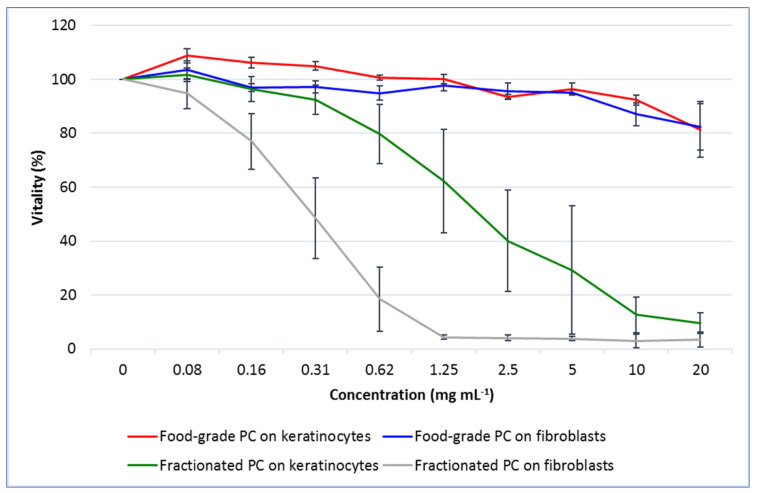
The vitality, expressed as percentage (mean ± SD), of cultured human dermal fibroblasts and keratinocytes treated for 24 h with increasing concentrations of a commercial phycocyanin (PC) and a PC fractionated from *Arthrospira platensis* as assessed with colorimetric cytotoxicity assay (MTS test).

Results of the MTS assay are shown in Figure 3. The commercial PC was nontoxic up to the maximum tested dose of 10 mg mL^−1^, which induced a decrease in cell viability still lower than 20% on both fibroblasts and keratinocytes. Differently, the fractionated PC induced a decrease in cellular viability greater than 20% already at concentrations of 0.16 and 0.62 mg mL^−1^ for fibroblasts and keratinocytes, respectively. Based on these results, for the antioxidant efficacy test, the nontoxic doses were set at 10 mg mL^−1^ for the commercial and at 0.08 and 0.30 mg mL^−1^ for the fractionated PCs for fibroblasts and keratinocytes, respectively.

### 2.5. Oxidative Status

#### 2.5.1. Chemical ROS Induction

**Figure 4:**
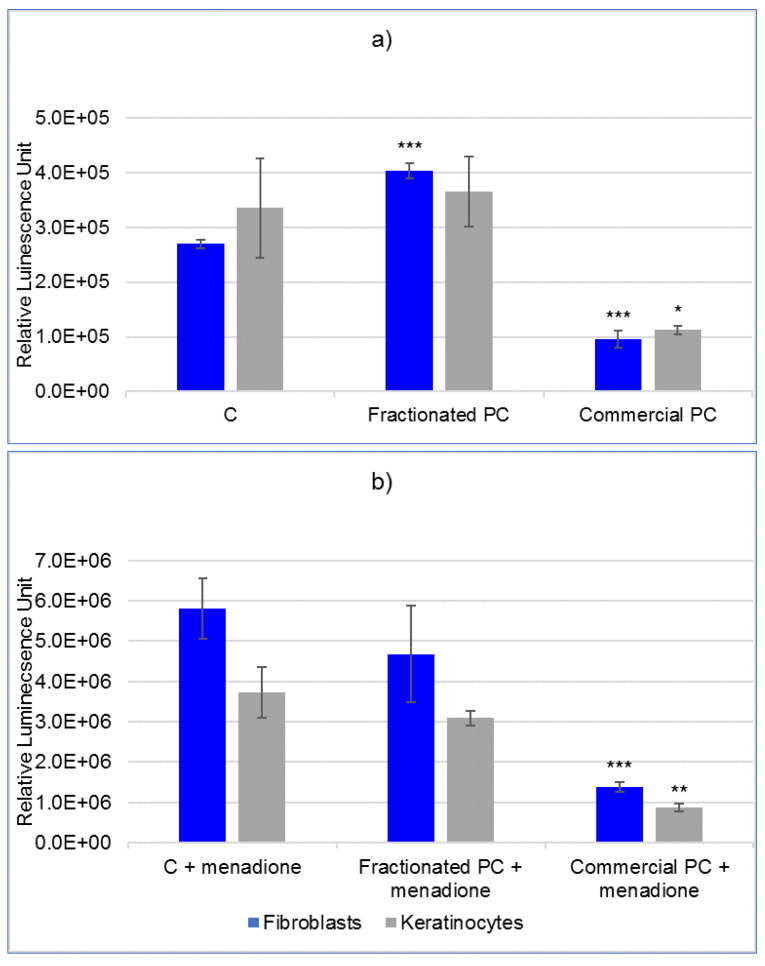
The quantification of reactive oxygen species, expressed as relative luminescence units (mean ± SD), in human fibroblasts and keratinocytes. Cells were incubated for 24 h with culture medium (C) as the control in the presence of a commercial phycocyanin (PC) and a phycocyanin fractionated from *Arthrospira platensis*: (**a**) not treated with the prooxidant menadione, (**b**) treated with menadione. Statistical analysis was conducted with Student’s *t*-test by comparing each treatment with control cells; the level of significance set at (***) *p* < 0.005, (**) *p* < 0.01 and (*) *p* < 0.1.

The results for the quantification of ROS in fibroblasts and keratinocytes prior to and after menadione-induced oxidative stimulus are reported in Figure 4a,b. Regarding the commercial PC, a statistically significant (*p* < 0.01) lower ROS level was recorded in the two cell types both prior to and after the pro-oxidizing treatment compared with the respective control cells. Differently, the fractionated PC not only did not counteract the ROS increase upon menadione treatment on both cell lines, but fibroblasts underwent a slight but statistically significant (*p* < 0.01) increase in the level of ROS even in the absence of prooxidant stimulus, in contrast with controls.

#### 2.5.2. Physical ROS Induction

**Figure 5:**
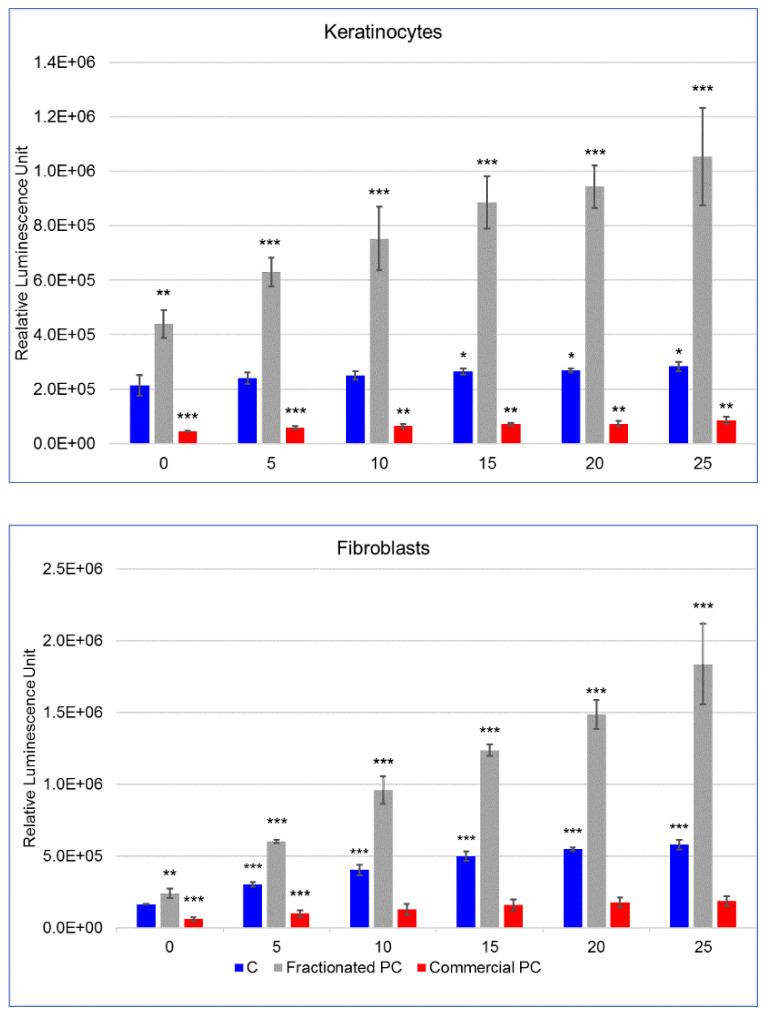
The quantification of reactive oxygen species, expressed as relative luminescence units (mean ± SD) in human keratinocytes and fibroblasts. Control (C) cells and cells incubated for 24 h with a commercial phycocyanin (PC) and a PC fractionated from *Arthrospira platensis* were exposed to UVA for 5, 10, 15, 20, 25 min. Statistical differences were evaluated via Student’s *t*-test by comparing each treatment with control cells not exposed to UVA (0 min); the level of significance set at (***) *p* < 0.005, (**) *p* < 0.01 and (*) *p* < 0.1.

The results of the ROS quantification in fibroblasts and keratinocytes after UVA exposure are shown in Figure 5. With regard to control fibroblasts, a constant significant (*p* < 0.001) increase of ROS was reported already after 5 min exposure when compared with not-irradiated cells. Moreover, in the cells not exposed to UVA, significantly different levels of ROS were shown for cells pretreated with PCs, higher (*p* < 0.01) for the commercial and lower (*p* < 0.001) for the fractionated PCs, when compared with the control.

The production of ROS was further increased by the fractionated PC during the subsequent exposure to UVA, while the commercial PC reduced ROS level even after 5 min UVA exposure compared with the not-exposed control cells. The protective effect of this PC was maintained throughout the following 25 min irradiation by ensuring intracellular levels of ROS not significantly different from those of the not-exposed control cells. With regard to keratinocytes, although the general picture was similar, some differences emerged. As first, these cells showed a nonsignificant increase in ROS compared with controls after 10 min of UVA exposure that became statistically significant only during the next 15 to 25 min. The fractionated PC also showed a considerably significant (*p* < 0.001) prooxidant effect in conjunction with UVA exposure on keratinocytes. The commercial PC was also highly protective on this cell line, where the ROS level was kept constantly lower (*p* < 0.001) in the irradiated cells than in the nonirradiated ones.

## 3. Discussion

The increasing interest in green chemistry has pushed the cosmetics industry toward the search of natural sources for innovative ingredients. In this respect, microalgae are a huge reservoir for the supply of bioactive molecules such as antioxidants, pigments, unsaturated lipids and vitamins [19]. As such, they have gained considerable importance for the development of cosmeceuticals due to their beneficial effects and therapeutical value for skin health [15,20]. In addition to its antioxidant properties, the extracts of Spirulina exhibit a bright color. In particular, with maximum absorption in the red–orange spectrum (580–680 nm) and a marked fluorescence emission at about 650 nm, PCs could serve as an attractive natural blue food dye [21] of potential interest for the cosmetics industry. Furthermore, technological advances allow increasing possibilities for extracting bioactive molecules from microalgae and other natural matrices through increasingly green and efficient processes [22]. In this regard, the results of the ORAC assay showed that the fractionated PC extracted by a relatively simple and green method had an antioxidant capacity almost three times higher than that of the commercial one. On the other hand, the cytotoxicity test results evidenced a high tolerability of the commercial PC on both fibroblasts and keratinocytes, whereas a much more marked toxicity was highlighted for the fractionated one.

As for this outcome, the two types of PCs deserve some considerations. First, it must be emphasized that the toxicity of PCs intended for food consumption has usually been evaluated in vivo on experimental animals. The results of our work clearly demonstrated that a commercial PC for food use could be safely administered to two human skin cell lines up to the remarkable dose of 20 mg mL^−1^. In this respect, some authors have recently shown that concentrations between 15.6 and 111.7 mg L^−1^ of this natural pigment were sufficient to replace traditional synthetic blue dyes in beverages [23]. This result provides an impressive range of shades for formulations in cosmetics even where extreme intense tones are desired. Differently, research on PCs of higher purity has more often focused on their biological activities of interest for the nutraceutical or medical sectors rather than for cosmetics [3]. For example, PCs of variable levels of purity have been tested on several malignant, non-tumorigenic and primary cell lines at concentrations hundreds or thousands of times lower than the 10 mg mL^−1^ at which we tested the commercial PC [24]. Therefore, in-depth studies on the real potential of low purity but economically advantageous PCs are required. In this respect, the results of our work have shown that the fractionated PC, which was of low-medium purity with respect to the highest reagent-grade category, had about 100 times higher toxicity compared with the commercial PC. Additionally, the fibroblasts of the connective tissue were almost three times more susceptible to the keratinocytes of the epidermis as shown by the concentration of PC, which caused the reduction of cell viability of about 20%.

The results relating to the protective efficacy of PCs against chemical and physical oxidative stimuli have revealed superiority of the commercial over the fractionated PC. This finding is evidently the direct consequence of the toxicity thresholds, which dictated the subsequent concentrations of use. In fact, while the fractionated PC showed an ORAC antioxidant potential approximatively three times higher than the commercial PC, its protective potential could only be assayed on cells at very low concentration. Nevertheless, despite the low doses, this PC showed an unexpected prooxidant activity in particular in fibroblasts even in the absence of external prooxidant stimuli. Otherwise, for keratinocytes, the prooxidant activity was observed in the experiment with UVA irradiation already in cells not directly exposed. The reason for this is most likely that polystyrene does not shield UVA [25]. Therefore, after exposing the plates, the cells artificially shielded by the protective film still underwent a partial stimulation. This prooxidant effect, defined as paradoxical, has been repeatedly documented for other well-known antioxidants such as vitamin C, vitamin E, its water-soluble analogue Trolox and different classes of polyphenols [26,27]. However, and in particular for the latter, objective confirmations of their real antioxidant potential to cells are still under debate considering that they have the ability to oxidize various molecules present in classic cell culture media, thus causing an abiotic production of reactive molecules and possible misinterpretation of results [28]. Regarding this, to the best of our knowledge, this is the first clue obtained directly on a cellular model that would suggest the inclusion of PC in the list of antioxidant compounds with possible paradoxical prooxidant effects if administered to cells in specific doses. To allow for further comparisons on this issue, it has been reported that crude highly fractionated PC administered at 25 µg mL^−1^ for 24 h was able to protect fibroblasts from oxidative stress induced by H_2_O_2_ [29]. Therefore, in a subsequent experiment (data not shown), we tested the fractionated PC at two concentrations of 25 and 10 µg mL^−1^. However, lowering the PC concentration inhibited the prooxidant as well as the protective effects. Differently, the commercial PC proved to be highly efficient in preventing the increase of ROS on the two skin cell lines against both chemical and physical stimuli and in reducing their levels that were already in unexposed cells. With particular regard to the effect against solar radiation, this result is in line with what has recently been published about the protective capacity of a very high-purity PC on human keratinocytes exposed to UVB [30]. However, and different from UVBs, which in the cited work depressed cell viability in a dose-dependent manner, the results of our present work revealed that UVA causes the rapid rise of ROS in fibroblasts but is tolerated by keratinocytes up to 25 min exposure. This result is in line with previous findings [31] that indicate the greater natural predisposition of keratinocytes over fibroblasts to repair DNA damage induced by sun exposure, against which keratinocytes represent the first defense. This evidence is not, however, completely reassuring in the case of UVAs as they have the ability to penetrate and reach the deeper layers of the dermis where the less resistant fibroblasts reside [6]. At the same time, it is necessary to reiterate the extreme importance of identifying more molecules, possibly of natural derivation, capable of preventing the alterations underlying cell damage before the cell runs out of its defensive resources. In fact, as clearly demonstrated [32], chronic UVA exposure can induce apoptotic resistance and malignant transformation in human keratinocytes once the resistance threshold is exceeded.

Because PC is a blue pigment–protein conjugate that easily undergoes denaturation following insults of various natures, several authors investigated the most promising conditions for safeguarding its functionality [33]. Among others, although heat is one of the main physical causes responsible for its degradation that limit its wider use in the food sector, solar radiation probably represents its greatest obstacle for the development of cosmetic preparations. In this work, we have verified that about 50% of the molecule was lost after twenty-five min direct exposure to UVA lamp. In this respect, keeping PC under acidic conditions, or even in highly viscous matrices containing sugars or hydrocolloids, such as beet pectin and guar gum [34], was demonstrated to increase its stability in the presence of heat and light. This information is interesting considering that topical cosmetics are usually formulated at pH 5 precisely to respect the physiology of the skin. Moreover, widely available sunscreens can help to preserve the functionality of PCs in an effective way [5], as indeed do the secondary metabolites, which act as natural sunscreens just for this purpose [35].

## 4. Materials and Methods

### 4.1. Materials

Raw Spirulina microalgae were purchased at a local private company (Salera Soc. Agr. s.s., Castelvisconti, CR, Italy) and a PC for food use was obtained through commercial distribution web channels at Fluxias (GmbH, Horgenzell, Germany). Cell lines were purchased at Caltag Medsystems (Buckingham, UK). In detail, early passage human dermal fibroblasts (ZHC-5301) were propagated no more than 15 times, while immortalized human keratinocytes were authenticated after extensive use (ID: 11107724332, Eurofins Genomics Europe Applied Genomics GmbH, Milano, MI, Italy). 

### 4.2. PC Extraction and Primary Purification

PC was extracted from freeze-dried Spirulina using a minor modification of a method reported in the literature [36]. Briefly, 5 g lyophilized Spirulina biomass was suspended in 500 mL 0.01 M phosphate buffer (0.15 M NaCl, pH 7.0), treated by sonication (Bransonic M3800H-E, Emerson, Danbury, CT, USA) for 15 min and left overnight under stirring. The mixture was then centrifuged (10,000× *g*) for 20 min at 4 °C. Thereafter, the blue supernatant was fractionally precipitated at 12,000× *g* for 30 min at 4 °C with ammonium sulfate at 15% and 45% (weight/volume). The first 15% precipitated fraction, containing chlorophylls, was discarded, while the 45% fraction rich in PC was resuspended in a small volume of phosphate buffer and fractionated through a tubular dialysis membrane (Spectra/Por 3.5 KDa, Spectrum) for 3 days against distilled water [36,37]; the filtrate was finally freeze-dried using a vacuum freeze-dryer for 3 days at −10 °C (model D01, Edwards, Cinisello Balsamo, MI, Italy).

### 4.3. Characterization of PCs

Both commercial and fractionated PCs were suspended in phosphate buffer to a final concentration of 0.6 mg mL^−1^. The purity of PCs was spectrophotometrically (LAMBDA 365 UV/Vis, Perkin Elmer) estimated as the ratio between the optical density relative to the absorption peak of phycocyanin (A_620_) and that of total proteins (A_280_), and absorption spectra were determined by scanning in the range 300–700 nm.

### 4.4. High-Performance Liquid Chromatography

HPLC analysis was performed on a chromatograph equipped with an LC 250 binary pump and a DAD 235 detector (Perkin Elmer, MI, Italy) using a modification of methods reported in the literature [17,37,38]. Chromatographic separation was achieved using the Discovery Wide Pore C5 column (Supelco, Sigma-Aldrich, St. Louis, MO, USA) with a mobile phase consisting of solvent A (acetonitrile containing 0.05% trifluoroacetic acid) and solvent B (water containing 1% methanol and 0.05% trifluoroacetic acid). Before injection, commercial and fractionated PC solutions were filtered through a syringe filter with a nylon membrane (0.2 µm, Nalgene, Milano, MI, Italy). An amount of 20 microliters of methanolic sample solution (1 mg mL^−1^) was injected, and compounds were eluted at 1.0 mL min^−1^. Chromatographic runs were carried out starting from 20% of solvent A for 1 min followed by a linear gradient to 85% of A over 30 min. Detection was set at 620 nm.

### 4.5. Evaluation of the Oxygen Radical Antioxidant Capacity (ORAC)

The ORAC evaluation of PCs was performed using the ab233473 Assay Kit (Abcam, Cambridge, MA, USA) according to the manufacturer’s recommendation. A standard curve was set up with 25 µL Trolox (0.2 mM) in a 96-well black plate using the following concentrations: 50, 40, 30, 20, 10, 5, 2.5, 0 µM in diluent. Test sample PCs were set up in triplicate using 10 mg mL^−1^ and 3 successive dilutions in the same diluent, as follows: 1:10; 1:100 and 1:1000 (*v*/*v*). Blank samples were provided using only diluent. Thereafter, 150 µL of fluorescein solution was added to each well and incubated for 30 min at 37 °C. Twenty-five µL of the free radical starter solution was poured into each well, and fluorescence was immediately read with a multiplate reader (Ex/Em = 480/520 nm). Readings were taken every 5 min for a total of 60 min. The area under the curve (AUC) was calculated at each concentration of Trolox, test and blank samples, and net AUC values were obtained by subtracting the blank AUC. Net values were plotted on the *y*-axis versus the Trolox antioxidant standard concentration on the *x*-axis. The Trolox equivalents (TE) of test samples were obtained using the standard curve, and the ORAC results were expressed as TE (µM) per 100 g of sample.

### 4.6. Assessment of PC Photodegradation

In order to quantify the extent of PC degradation upon UVA irradiation, PCs aliquots were dissolved in cell culture medium, as described below, in plastic cuvettes and exposed to UVA radiation (366 nm) at a total dose of 7140 mJ cm^−2^ administered during 5 min under an UV lamp (Camag, Muttenz, Switzerland). The optical density was assessed every 5 min immediately after exposure using a spectrophotometer (7310, Jenway, Stone, UK) at the wavelength of 620 nm, corresponding to the PC absorption peak.

### 4.7. Evaluation of PCs on Cellular Models

#### 4.7.1. Cell Cultures

Cells were cultured in 100 mm petri dishes in 12 mL Dulbecco’s modified Eagle’s medium (DMEM) (Sigma Aldrich, Rome, Italy) supplemented with 10% fetal bovine serum (Sigma), penicillin (100 UI mL^−1^) and streptomycin (100 µg mL^−1^) at 37 °C in 5% CO_2_ humidified atmosphere. For treatments and related analyses, cells were harvested by trypsinization and used as appropriate.

#### 4.7.2. Cytotoxicity Test

First, cells were assayed by the MTS [3-(4,5-dimethylthiazol-2-yl)-5-(3-carboxymethoxyphenyl)-2-(4-sulfophenyl)-2*H*-tetrazolium, inner salt; U.S. Pat. No. 5,185,450] test to detect any potential cytotoxicity of the PCs. Fibroblasts and keratinocytes were seeded into 96-well plates (Sarstedt, Verona, VR, Italy) at 10,000 cells/well in 100 µL DMEM and grown for 24 h until adhesion. Thereafter, the medium was removed by overturning the plates and completely renewed with the same medium containing increasing doses of PC as follows: 0 (control), 0.08, 0.16, 0.32, 0.64, 1.25, 2.5, 5, 10 and 20 (mg mL^−1^). Cells were incubated for 24 h, and viability was assessed with the CellTiter 96^®^ AQueous One Solution Cell Proliferation Assay (Promega Corporation, Madison, WI, USA). Briefly, 20 µL MTS solution was added to each well and incubated for 3 h. Absorbance was then recorded at 490 nm using the GloMax^®^ Explorer System (Promega). Values were corrected by subtracting the background absorbance detected in wells containing only DMEM and PC at corresponding doses. The results were expressed as the percentage variation with respect to control cells at 100%.

#### 4.7.3. Oxidative Status

The evaluation was performed using the ROS-Glo™ H_2_O_2_ Assay (Promega), which measures the content of hydrogen peroxide (H_2_O_2_) in the culture medium. Cells (5000 cells/well) were plated in 80 µL DMEM in 96-well opaque walled white plates (Greiner Bio-One Italia S.r.l., Cassina de Pecchi, MI, Italy) and incubated to allow complete attachment. The day after, PCs were added at a nontoxic concentration and incubated for 24 h. Thereafter, 20 µL H_2_O_2_ substrate solution was added to each well (25 µM final concentration) and incubated for 1 h. Then, 100 µL ROS-Glo™ Detection Solution was distributed in each well and incubated for 20 min. Luminescence was finally read using the GloMax^®^ Explorer System, and results were expressed as relative luminescence units (RLU). In order to evaluate the protective effect of PCs against stimuli of a chemical nature, 1 hour before reading the luminescence, 50 µM menadione was added to control and PC-treated cells. To induce ROS production by physical means and to assess the residual protective efficacy of PC upon its decay, cells cultured in 96-well plates were UVA irradiated at a dose of 7140 mJ cm^−2^ at successive intervals of 5 min. Because treatment was conducted outside the incubator, in order to standardize the conditions between samples, control and treated cells were set up in a single plate, and the wells not intended for exposure to UVA rays were shielded with adhesive aluminon strips.

### 4.8. Statistical Analysis

The evaluations included a minimum of three replicates per test using three sample series per replicate. The results of the PC photodegradation, MTS and ROS assays on the cellular models are expressed as mean ± standard deviation. Otherwise, the ORAC values are expressed as mean ± standard error of the mean (SEM) according to the U.S. Department of Agriculture (USDA) requirements for the implementation of the National Nutrient Database for Standard Reference reporting the oxygen radical absorbance capacity of foods and spices [39]. Differences between RLUs relative to ROS estimation were statistically evaluated by Student’s t-test after logarithmic normalization, and values in tables are presented as raw data.

## 5. Conclusions

A commercial PC for food use that is easily available and with a very high safety threshold has shown a strong ability to prevent the production of ROS, one of the main causes of cell alteration, after the induction of chemical and physical (UVA rays) stress on human fibroblasts and keratinocytes. These results, together with its great coloring power, make this molecule an excellent candidate for formulations capable of satisfying various marketing-related aspects ranging from cosmetic appeal to functional claims. Of course, in the case of preparations of potential protective or soothing creams designed for sun exposure, given the rapidity with which PC is degraded by UVA, the introduction of appropriate shielding molecules becomes mandatory to safeguard and prolong its effectiveness. On the other hand, regarding the possibility of using PCs obtained directly from extraction from algal matrices, the advice is to push the purification processes in favor of PCs over other proteins and possible general contaminants also taking into account the necessary additional costs. To achieve this, the application of further purification processes, such as chromatographic columns, becomes necessary in addition to dialysis with tubular membranes. However, to better elucidate the protective and paradoxically prooxidant mechanisms of PCs of different purity on UV-induced skin damage, more in-depth studies at the cellular level are required. In particular, studies on the combined effects of PC and molecules capable of shielding UVs while preserving their antioxidant activity would be of great interest.

## Data Availability

Data are contained within the article.

## Sample Availability

Not available.
